# Examination of Chinese Gambling Problems through a Socio-Historical-Cultural Perspective

**DOI:** 10.1100/tsw.2010.167

**Published:** 2010-09-01

**Authors:** Samson Tse, Alex C.H. Yu, Fiona Rossen, Chong-Wen Wang

**Affiliations:** ^1^ Department of Social Work and Social Administration, University of Hong Kong, Hong Kong; ^2^ Applied Learning Technologies Institute, Arizona State University, Tempe, AZ, USA; ^3^ Department of Social and Community Health, Tamaki Campus, School of Population Health, University of Auckland, New Zealand; ^4^ Centre on Behavioural Health, University of Hong Kong, Hong Kong, Hong Kong

**Keywords:** addiction, culture, gaming, Chinese history

## Abstract

The aim of this review is to highlight emerging trends about Chinese people and gambling addiction over the last 15 years, and to provide a discourse on the potential link between gambling and Chinese culture and history. The authors reported on the phenomenon of gambling among Chinese people using relevant research studies and reports and traditional Chinese literature. Chinese people have elevated levels of gambling addiction compared to their Western counterparts. These elevated rates are coupled with the rapid expansion of gambling venues within the Pan-Pacific region. While there is an accumulated body of research on Chinese and gambling, a systematic cultural analysis of Chinese gambling is still under development. We undertook a brief comparison between two ancient civilizations, China and Rome, in order to gain better understanding about gambling among Chinese people. To effectively deal with gambling addictions among Chinese people, it is imperative to develop culturally responsive interventions.

## INTRODUCTION

For the last 10–15 years, there has been a surging interest in studies of gambling behaviors among different ethnic groups, including Asian refugees and Hispanics in the U.S.[1,2,3]; Chinese in Canada[4,5,6,7]; Maori (indigenous people of New Zealand) and Chinese in New Zealand[8,9,10]; and Arabic, Chinese, Greek, and Vietnamese in Australia[11,12,13,14]. Closer examination of these published studies shows that a considerable amount of work has concentrated on Chinese people and gambling. Although some studies have investigated the prevalence rate and relevant risk factors of problem gambling among ethnic Chinese[4,5,6,7,11,15,16], and the links between gambling and traditional Chinese culture[8,9,10], few studies have examined gambling behaviors among the Chinese from a social-historical-cultural perceptive. In this paper, some emerging trends about gambling among the

Chinese in the Pan-Pacific region are examined, followed by the comparison of Chinese history with another ancient culture, Rome, and a discussion on gambling in current Chinese societies. The paper concludes that there is a need for a culturally specific intervention strategy to address problem gambling for Chinese people.

## ELEVATED RATES OF PROBLEM GAMBLING AMONG CHINESE PEOPLE

"Pathological gambling" refers to a condition in which individuals meet five or more Diagnostic and Statistical Manual IV (DSM-IV) criteria[17]. A person suffering from pathological gambling shows persistent and recurrent maladaptive gambling behavior, resulting in dysfunctions in the areas of work, studies, and social and family relationships. Whereas "problem gambling" refers to a condition in which a person meets three or four DSM-IV criteria, and the person has the following problems: high rates of gambling-related fantasy, lying, using gambling to escape, and preoccupation about gambling, "disordered gambling" is used to describe the combination of problem and pathological gambling. Disordered gambling is associated with substantial interpersonal, financial, and legal difficulties, coupled with increased levels of substance abuse, mood and anxiety disorders, and suicidality in some cases[3]. There is growing evidence to suggest that Chinese people exhibit elevated levels of problem gambling; estimates range between 2.5 and 4% of the adult Chinese population compared to between 1.5 and 2% in Western populations[18]. In the context of the present paper, Western populations refer to English native speakers and Europeans in the U.S., U.K., Canada, Australia, and New Zealand. A population-based study in Hong Kong found that up to 6% of the respondents met the diagnostic classification of probable problem and pathological gambling[19], whereas nationwide surveys in the U.S.[20] and New Zealand[21] invariably show lower rates of problem gambling for primarily Western participants (see Table 1).

**TABLE 1**
**Comparison of 12-Month Prevalence of Disordered Gambling in the U.S., New Zealand, and Hong Kong**

| | Disordered Gambling | Year of Study | Instruments Used |
|---|---|---|---|
| U.S.[20] | 1.8% | 2001 | Diagnostic Interview Schedule |
| New Zealand[21] | 1.2% | 2002/03 | South Oaks Gambling Screen |
| Hong Kong[19] | 6.0% | 2003 | DSM-IV |

It is likely that a small portion of the variation in these problem rates is attributable to the use of different instruments. However, the measurement tools used in all three of these surveys are based on the assessment principles employed by the DSM-IV and have been demonstrated as valid and reliable measures of problem gambling. Arguably, it is possible that the problem gambling rates for Chinese communities are under-reported because of the fear of losing face under the influence of Confucianism within Chinese culture[5].

Not only do Chinese people have high rates of problem gambling in predominantly Chinese urban cities, the Chinese as an ethnic minority group also have a relatively higher prevalence rate than their counterparts in the host societies. Some studies have found that problem gambling rates in Chinese communities are between 1.5 and 5 times higher than those of local people[12,20,22]. For instance, the 2001/2002 National Epidemiologic Survey on Alcohol and Related Conditions confirmed that Asians, Blacks, and Native Americans have a significantly higher lifetime prevalence of disordered gambling than Whites by using the DSM-IV assessment on pathological gambling (see Table 2)[3]. Furthermore, there is

**TABLE 2**
**Comparison of Lifetime Prevalence of Disordered Gambling among Four Populations in the U.S.[3]**

| | Disordered Gambling (Confidence Level) |
|---|---|
| Native Americans/Asian | 2.3% (1.5–3.3)* |
| Blacks | 2.2% (1.8–2.6) |
| Hispanics | 1.0% (0.8–1.4) |
| Whites | 1.2% (1.0–1.3) |

* The prevalence of disordered gambling among Native Americans/Asian differed significantly from Whites ($p$ = 0.01) and Hispanics ($p$ = 0.007).

emerging evidence to suggest that Chinese immigrants (e.g., from Hong Kong, mainland China) may develop higher rates of problem gambling with increased years of residency in newly adopted countries[4,5,23]. Further evidence of the disproportionate effects of gambling on Chinese people has been gathered in New Zealand. First, the median money lost (in the 4 weeks prior to seeking help) by Asian clients (the majority of whom were Chinese) who presented to problem gambling intervention services in 2006 was NZ$3,000 (or approximately US$1,965) compared to NZ$800 (or approximately US$525) for non-Asian clients. Second, Asian clients accounted for only 7% of the total New Zealand new face-to-face clients in 2006, while accounting for 42.7% of the reported losses[22].

Although the reported prevalence rates of problem gambling among different populations or cultural groups may not be conclusive because of different operational definitions of problem gambling or use of measurements, the aforementioned figures do raise the alarm about the seriousness of the problems among Chinese people. In order to better understand Chinese gambling problems and develop culturally responsive intervention strategies for Chinese affected by problem gambling, the authors suggest an examination of Chinese gambling behaviors from socio-historical and cultural perspectives.

## SOCIO-PERSPECTIVE: EXPANSION AND PROLIFERATION OF GAMBLING IN THE PAN-PACIFIC REGION BETWEEN 1995 AND 2010

Gambling includes a wide range of forms, such as lotteries, betting on horse and dog (greyhound) races, betting on sports (e.g., football betting), mahjong houses, and casino games. In practice, casino games are favored by many Chinese patrons because of the variety of games, lack of time limits, attractive promotion programs, availability of credit, and unlimited odds of betting[18]. Currently, legalized gambling, such as lotteries, has been introduced into most countries or areas in the Pan-Pacific region, including China. Although casino games remain illegal in mainland China and Hong Kong, they have been legalized in Macau, Taiwan, Singapore, the Philippines, and South Korea.

The present authors tracked the change in the number of casino venues in the Pan-Pacific region between 1995 and 2010 through the revision of information available on the Internet and hard copies of relevant documents. Fig. 1 shows the numbers and location of casinos in 1995 (as depicted by yellow dots) and 2010 (depicted by black dots) in the region. It can be seen that a total of 16 casinos existed in the Pan-Pacific region in 1995, a figure that has risen to a total of 77 in 2010.

There has been an almost fivefold increase in the number of casinos over the 15-year span between 1995 and 2010, a comparatively short period of time. Although a substantial proportion of the growth in casinos over the last 1.5 decades can be attributed to the recent introduction and expansion of the Western gambling industry in Macau, the number of casino venues has also increased significantly in other locations, such as the Philippines and South Korea. The high concentration of gambling venues in the Pan-Pacific region in the 21st century echoes very much what was said almost 50 years ago: “Southern

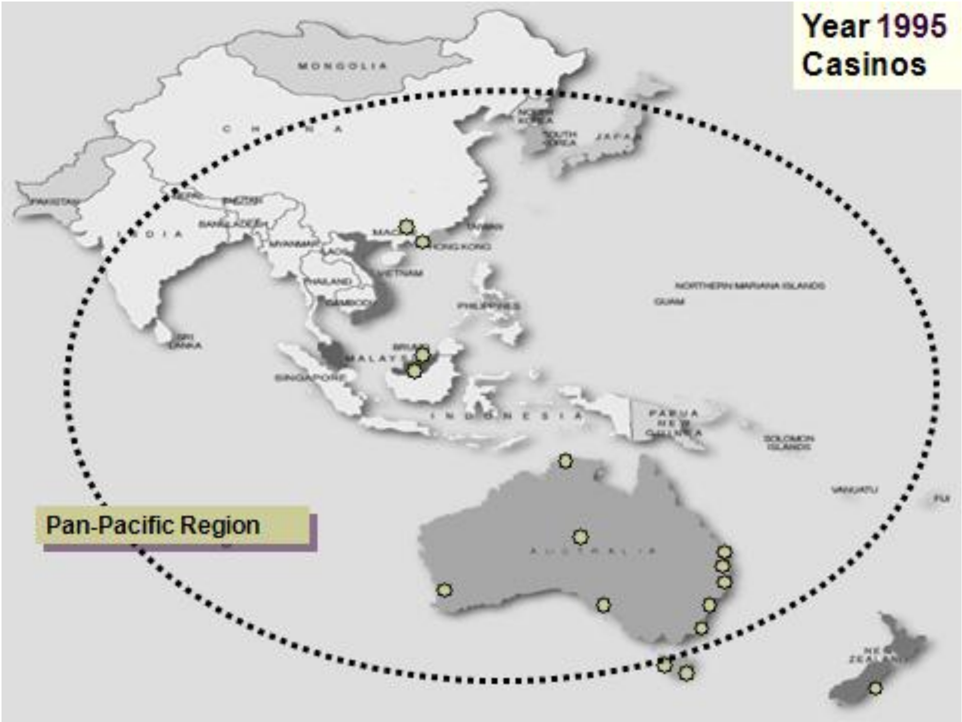

**A**

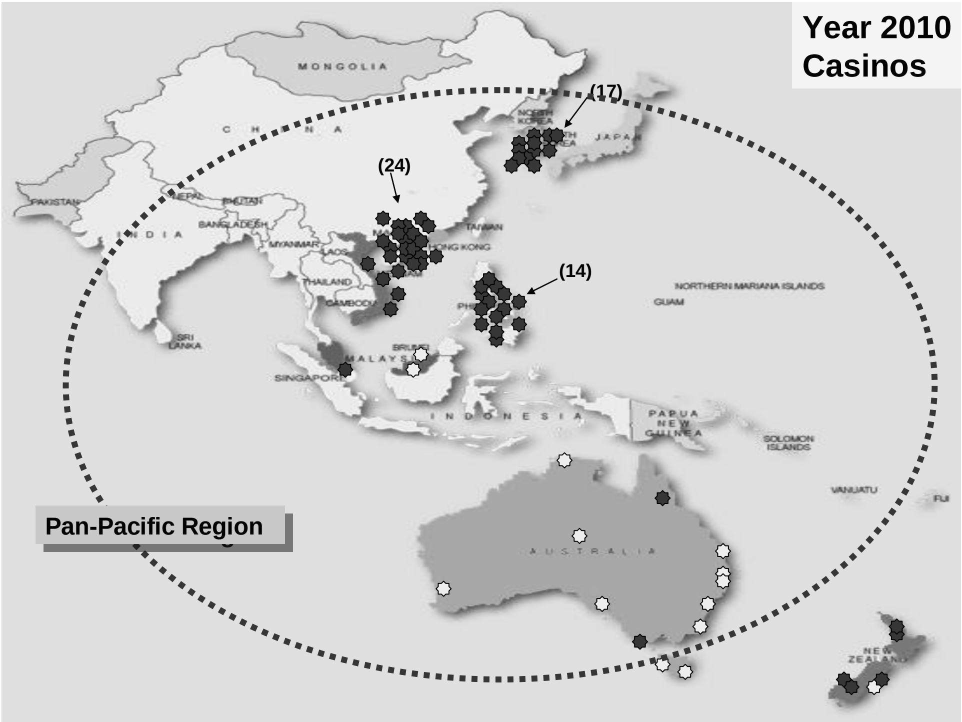

**B**

**FIGURE 1.** (A) Grey dots denote the number and location of the casinos in 1995 in the Pan-Pacific region; (B) black dots denote the number and location of additional casinos in the year 2010 in the Pan-Pacific region.

China and Southeast Asia is one of the most intensively gambling regions of the world" (Olmestead, 1962, cited in [24], p. 163). The authors contend that, first, this trend of intensified gambling activities will continue and that it is being reinforced by globalization, which is characterized by more affordable international travel both within the region and globally, and a strong economy in the Asian region despite the recent global economic crisis. Second, there is a rich body of research studies to suggest a positive relationship between proximity of casino venue and participation in gambling activities[25,26], thus it is plausible that the problem gambling rates in the Pan-Pacific region will be on the rise or remain at a relatively high level in the medium and long term.

## HISTORICAL AND CULTURAL PERSPECTIVE: GAMBLING SANCTIONED BY RULERS THROUGHOUT CHINESE AND ROMAN HISTORY

The first account of gambling in China could be found in the feudal states in the late Chou Dynasty (800–256 B.C.), although some researchers have pushed the origin even further back to the Shang (1700–1027 B.C.) and the Xia (2000–1500 B.C.) Dynasties[27]. Throughout these times, there was a variety of gambling methods, such as Chinese football, cock fighting, duck fighting, goose fighting, horse racing, and dog racing. In order to illustrate the unique historical nature of Chinese gambling, comparison was made to the history of Roman gambling. As with China, different gambling games spread throughout almost every corner of the Roman Empire, including in the different public baths, the circuses, the taverns, and the imperial forums. Roman gambling activities included Tali and Tesserae, which were two types of dice games. The Tesserae dice had six sides, but the Tali dice had four sides only and the numbers were 1, 3, 4, and 6. Another example is the Duodecim Scripta, a word game that resembles Backgammon.

Throughout Chinese history, only the Sung (960–1279), Yuan (1279–1368), and Ming (1368–1644) Dynasties were serious about outlawing gambling. However, legal actions against gambling only occurred in the beginning eras of those dynasties. After a while, not only were the laws no longer enforced, but some rulers themselves also practiced gambling. Zhu Yuan-zhang (1328–1398), the first Emperor of Ming, planned to launch bold construction projects and thus outlawed gambling in order to redirect manpower from this time-consuming hobby to more productive activities. However, Zhu failed to set a good example as he frequently gambled with his subordinates. A number of very similar incidences have been reported in Roman history. At one time, the only occasion during which the Roman population could legally gamble was the carnival feast known as Saturnalia. However, Emperor Augustus, as an obsessive gambler, did not limit his gambling to Saturnalia only. Emperors Nero, Claudius, and Commodous were also lovers of gambling, and Commodous even "turned the imperial palace into a regular Monte Carlo" ([28], p. 104). As a result, similar to the Chinese history, the bad examples of some of the imperial courts or Emperors led to the practical abolition of the aforementioned antigambling laws.

Following on from the middle Ming period in Chinese history, many Emperors and civilians were obsessed with gambling. After the Qing (1644–1911) army forced the Ming government to retreat to South China, Ming's Chinese began to be fond of another form of gambling, cricket fighting, despite the Qing army posing a serious threat to the remaining Ming's region. Thus, some historians attributed the fall of Ming to paper mahjong and the fall of South Ming to cricket fighting[29]. Similar to the Chinese history of gambling, Romans were aware of the damages caused by gambling and, thus, antigambling laws were passed from time to time, but, generally speaking, these laws failed to contain the gambling-related harms. Cato attempted to restrict gaming by dice to adults only because it was thought that the young should be out in the fields practicing their military skills. For military training, there was a senatorial decree banning wagers except on one's own skill in throwing the spear, running, jumping, wrestling, or boxing[30]. However, it was suspected that these restrictions were neither enforceable nor enforced[30]. The laws entitled *Lex Cornelia*, *Lex Publicia*, and *Lex Titia* also forbade gambling of dice, and the offenders could be sent to jail or fined. The law named *Lex Talaria* prohibited the gambling of dice specifically at meal times, but it was ineffective. Cicero mentioned that some gamblers repeated the same offence over and over[28]. Additionally, Roman law did not recognize debts resulting from gambling. If a Roman citizen lost money to a slave or to a minor from gambling, the court would rule the winning against the master of the slave or the parent of the minor. Conversely, if the slave or minor were the loser, suit might be brought by master or parent to recover the sum. As part of the measure for problem gambling, Ulpian proclaimed that it was illegal for anyone who used force to compel another to play dice[30].

## DIFFERENT PATHS TOWARDS GAMBLING: CHINESE AND ROMAN GAMBLING HISTORY

Given the startling similarities between the Chinese and Roman histories of gambling, one would ask why the two cultures turned out to have a quite different profile in terms of participation in gambling activities and problem gambling in the 21st century. Not only are patrons of Chinese descent very noticeable in most gambling venues, their level of disordered gambling is found to be higher compared to their European counterparts who had been influenced by Roman culture historically. The present authors propose three possible explanations.

First, there is a long history of Chinese rulers and governments using gambling as a means of financial support. In China, after the Opium War in 1840, Qing Emperors lost almost every single war to the Western powers. Because Western nations demanded a huge sum of reparations, the Qing government legalized gambling and sold casino licenses in order to collect sufficient funds for paying reparations. Some local officials disapproved of this short-sighted practice and proposed banning gambling again, but this did not progress as the central government then asked the local governments to find alternative ways to collect the same amount of money if gambling were prohibited. Besides paying reparations, supporting the defense budget was another justification for legalizing gambling. In 1884, China sent troops to Vietnam to fight against the French. In order to increase the military expenditure for this venture, the Qing general used legalized gambling as a means of making quick money[29]. During the late Qing period, gambling was more widespread in Canton (Guangzhou), a subprovincial city and the capital of Guangdong Province, which was arguably the number one region in gambling across the entire nation; thus Canton was nicknamed "the nation of gambling". Furthermore, from the middle of the 1800s to the early 1900s, with the arrival of the Westerners (since 1842) and the establishment of Special Economic Zones, sovereignty from Chinese law was decreed and these zones were divided into various parcels governed by different countries. Shanghai became a magnet that attracted many Chinese gamblers who engaged in a variety of local and foreign games, including roulette, in its large gambling complexes[31]. Western-style casino games were also brought to Macau in the 20th century[32]. Later, gambling spread to Hong Kong. The gambling custom scattered to every Chinese community around the globe as Chinese emigrated to overseas countries, e.g., to California in the U.S. and from the 18th century, to Queenstown in New Zealand[29,33].

Second, to examine Chinese gambling successfully would require us to look beyond Chinese history. An increasing volume of studies has suggested that the Chinese tend to have a higher external locus of control than their Western counterparts regardless whether the research participants were university students or clients presenting to problem gambling services[31,34,35]. The higher external locus of control has a potential to lead to a higher illusion of control and distorted thinking, and may increase an individual's level of participation in gambling. If the gambler wins a lot of money, it will affirm the belief that he/she is destined to be a winner. However, if the person loses money, he/she would say, "One day my turn will come." Thus, it is often difficult for these individuals to stay away from gambling, making them vulnerable to problem gambling. Locus of control may explain why social gambling is a very common activity among Chinese societies. Superstition is another factor, indeed, that may increase the propensity for excessive gambling. Many Chinese believe that one's success and wealth depends on fate, luck, Feng Shui (certain architectural styles that could affect one's fortune), and/or accumulation of good deeds[30]. According to the *Analects of Confucius*, life and death are a matter of fate; riches and poverty depend on heaven. On the one hand, Chinese people admit that life is uncertain and events are driven by hidden fate and luck. On the other hand, for the Chinese, fate is not equated with passivity. Turning the tide of fortune, in Chinese thinking, is still possible as life events unfold. Some Chinese people are confident that they could change the course of fate and increase their chance of winning by practicing Feng Shui and/or seeking advice from fortune tellers and palmists. Most people believe that luck, destiny, and chance control their lives. Some Chinese believe that, through gambling, they can unmask their concealed destiny[5]. Although numerous past studies have indicated that superstitions, traditional values and beliefs, social class, socialization processes, peers, and family all influence and have impacts on one's level of participation in gambling, there has been very limited work to compare and contrast the impact between Chinese and non-Chinese cultures[16,36,37]. Therefore, no definite conclusion can be drawn.

Third, it was noted that gambling activities in the Roman Empire were not checked effectively until its end. Lancian (1892)[28] attributed this turnaround to the spreading of Christian values. Anderson (1997)[38] also asserted that Christian values counteract the gambling culture. In his view, the Bible urges humans to work creatively and for the benefit of others, but gambling promotes a "something for nothing" attitude. The Bible condemns materialism, but gambling promotes it. Using Weber's work on the role of the Protestant ethic in social control, Pontell and Geis[39] argued that Christianity provided some proscriptions against gambling in the European culture, while similar social-religious controls were not evident in the Chinese culture. In the modern context, there are a considerable number of Christian- or faith-based organizations that provide professional counseling services for individuals affected by problem gambling and their concerned significant others, e.g., Ray of Hope in Singapore, Industrial Evangelistic Fellowships in Hong Kong. It might be debatable whether the demise of gambling in the Roman Empire was due to the rise of Christianity or other causes; nonetheless, it is obvious that the two ancient civilizations, China and Rome, took different paths towards gambling.

## CURRENT GAMBLING PRACTICES ACROSS CHINA

All forms of gambling, including lotteries, were outlawed in mainland China for more than 3 decades following the foundation of the People's Republic of China in 1949. Due to the lack of a public welfare system, the first charity-based lottery in the mainland was issued in 1987. This was in response to the suggestion by the Ministry of Civil Affairs that this would enable the state to collect funds to support welfare establishment in mainland China[40]. In 1989, a sports lottery was introduced in China for the first time in an attempt to raise funds for the 11th Asian Games. This was followed in 1994 by the establishment of a nationwide sports lottery-selling network. Since then, the sports lottery has played a more and more important role in raising funds to help China's western and less-developed regions supplement the budget of major sports tournaments[41]. These two lotteries were soon competing to attract more customers. As such, lottery tickets became more alluring in the 1990s with bigger prizes, such as apartments, houses, cars, color TVs, and laundry machines. Prizes were often showcased in public arenas, usually at large outdoor plazas, to attract punters[40]. Today, the lottery in China is more widespread than ever before. Recent times have also seen a trial of modern horse racing betting in Wuhan City and the Chinese government plans to open hotel resorts and casinos on Hainan Island.

Along with legal lotteries, illegal gambling has proliferated in mainland China in the past 2 decades, resulting in a significant increase in the rates of problem gambling[42]. The gambling takes place in card and mahjong schools on street corners, in underground casinos in the cities, through unofficial lotteries in the countryside, and on websites catering to Internet gamblers. Around US$150 billion are wagered illegally each year in mainland China, approximately 10 times the amount of the two officially sanctioned lotteries in mainland China[42]. In recent years, illegal gambling has emerged as a serious social problem in mainland China. Increased income inequalities, progressive differentiation between rich and poor, and an illusion of getting rich quickly has driven some individuals to gamble problematically.

Unlike mainland China, gambling in Macau, an ex-Portuguese colony off the Southern coast of China, has been legalized and heavily promoted[32]. Gambling has been a significant contributor to the city's economy since the 1850s. The handover of Macau from Portugal to China in 1999 saw a boom in the number of casinos. Nowadays, Macau has become well known as the "Oriental Las Vegas". In addition to casinos, a number of other forms of gambling are also now available, including horse racing, greyhound racing, sports betting, and a number of lotteries[43]. In 2003, the Macao Gaming Inspection and Coordination Bureau reported that the gambling tax contributed 74% to the Macau fiscal revenue. In 2004, the percentage rose to almost 78% of the Macau total public revenue, which amounted to 15.2 billion Macau Patacas (or approximately US$1.9 billion). Macau has a population of 463,000 in 2010[44].

The gambling history of Hong Kong has many parallels to that of Macau, a city in close proximity. The only legal gambling provider in Hong Kong is the Hong Kong Jockey Club (HKJC). Horse racing commenced in Hong Kong in 1841 with the arrival of the British, and legalized gambling expanded with the introduction of the Mark Six lottery in 1975 and regulated football betting in 2003[45]. The HKJC claims to be the single largest taxpayer in Hong Kong, accounting for over 8% of all taxes collected in Hong Kong between 2006 and 2007. This amounted to over HK$12.6 billion/year (or approximately US$1.62 billion); Hong Kong has a population of 7 million in 2010[46]. The HKJC donates an average of HK$1 billion/year to charity and community causes, and compares itself to the Rockefeller Foundation in the U.S. In addition to these authorized modes of gambling, illegal gambling activities are offered by local bookmakers and unauthorized offshore operators in Hong Kong, including betting on overseas sporting events (e.g., the recent 2010 World Cup in Soccer) and races, as well as the Mark Six lottery. Although the proportion of people betting with illegal bookies has decreased in recent years, the scale of illegal gambling and its associated problems still deserve public attention[47]. Casino games are illegal in Hong Kong, but have become legalized in Taiwan and most recently Singapore. As a result, both places attract tourists from mainland China and other areas or countries[48].

## CONCLUSION

Overall, the influences on Chinese gambling are variable, but there is a possibility that socio-historical and cultural factors may have a considerable impact on the phenomenon. Although this paper review does not offer conclusive evidence on the links between those factors and Chinese problem gambling, there are important issues worthy of further discussion. The Chinese gambling problem should be a concern for social services and health professionals. Not only does problem gambling lead to individual loss of money and health, it also results in many social problems. For instance, numerous news or, indeed, scandals emerging from mainland China have illustrated some of the wider impacts associated with problem gambling: When a member of the government Party experiences an addiction to gambling, not only is their promising career ruined, but the reputation of the Communist Party is also damaged[49]. To date, there is a lack of empirical data or theory-driven studies on Chinese perceptions related to problem gambling and how interventions affect the well-being and recovery prospects of service users.

Previous studies and reviews on gambling among the Chinese concluded that individuals affected by problem gambling are reluctant to seek professional help[18,50]. Currently, the theories, assessments, and interventions developed in the West are still the dominant approaches for explaining the phenomenon of Chinese gambling and developing treatments for Chinese problem gamblers[18,51]. While cognitive-behavioral therapy is widely used in providing psychological interventions to problem gambling[50], this approach may not be effective without taking into account the attitudes, beliefs, and values within the context of different cultures. Thus, there is an urgent need for theory-based interventions specifically tailored to meet the needs of Chinese problem gamblers. Evidence is gathering that suggests that cultural differences exist in patterns of gambling (e.g., cognitive scheme, decision-making and risk-taking patterns) and general responses emerging in psychotherapy (e.g., interdependency among family, expectation for directive therapy) when compared with Western samples[18,52,53]. The examination in this paper of gambling behaviors from multiple perspectives, such as historical background, social environment, and locus of control, can assist with moving the focus on Chinese gambling so that it is more appropriate and compatible for use with Chinese people; moving beyond risk analyses to a focus on early help-seeking behaviors, fundamental character strengths, and personal inspirations, which act as protective factors and motivate one to refrain from excessive gambling. For instance, Shek[54] and colleagues[55] have developed a positive youth development framework to prevent problem gambling among Chinese adolescents. The key elements of the framework include promotion of bonding, resilience, social competence, emotional competence, cognitive competence, behavioral competence, moral competence, self-determination, spirituality, self-efficacy, beliefs in the future, development of a clear and positive identity, provision of opportunities for exercising positive behaviors, prosocial involvement, and prosocial norms.

Gambling has been performed by the Chinese people for thousands of years and has become a preferred social activity among various Chinese communities. To address gambling problems among the Chinese people, a culturally sensitive psycho-socio-spiritual strategy has to be considered. The directions for future research are: What are the specific strategies that have influenced the success of an intervention or counseling program for the Chinese and their concerned significant others affected by problem gambling? What are the medium- and long-term impacts of intervention programs on service users?

## ACKNOWLEDGMENTS

We gratefully acknowledge the contributions of anonymous reviewers whose inputs significantly improved the quality of this manuscript.

## References

1. Petry, N.M., Armentano, C., Kuoch, T., Norinth, T., and Smith, L. (2003) Gambling participation and problems among South East Asian refugees to the United States. *Psychiatr. Serv.* **54**, 1142–1148.
2. Wallisch, L. (1996) Gambling in Texas: 1995 Surveys of Adult and Adolescent Gambling Behavior. Texas Commission on Alcohol and Drug Abuse, Austin.
3. Alegria, A., Petry, N., Hasin, D., Liu, S., Grant, B., and Blanco, C. (2009) Disordered gambling among racial and ethnic groups in the US: results from the National Epidemiologic Survey on Alcohol and Related Conditions. *CNS Spectr*. **14(3),** 132–142.
4. Lee, B.K., Fong, M., and Solowoniuk, J. (2007) Transplanted lives: immigration challenges and pathological gambling among four Canadian Chinese immigrants. In *Proceedings of the Inaugural Asian Pacific Problem Gambling Conference 2005*: *Advances in Problem Gambling: Theory, Service and Research in the Asia-Pacific Region*. Shek, D., Yiu, T.I., and Chan, E., Eds. Tung Wah Group of Hospitals, Social Practice and Research Centre, and The Chinese University of Hong Kong, Hong Kong.
5. Sin, R. (1996) Gambling and Problem Gambling among Chinese Adults in Quebec: An Exploratory Study. Report prepared for Chinese Family Service of Greater Montreal.
6. Papineau, E. (2005) Pathological gambling in Montreal's Chinese community: an anthropological perspective. *J. Gambl. Stud.* **21**, 157–178.
7. Lai, D.W.L. (2006) Gambling and the older Chinese in Canada. *J. Gambl. Stud.* **22**, 121–141.
8. Dyall, L., Tse, S., and Kingi, P. (2009) Cultural icons and marketing of gambling. *Int. J. Ment. Health Addict.* **7(1),** 84–96.
9. Wong, J. and Tse, S. (2003) The face of Chinese migrants' gambling: a New Zealand perspective. *eGambling*. http://www.camh.net/egambling/issue9/opinion/wong/.
10. Volberg, R.A. and Abbott, M.W. (1997) Gambling and problem gambling among indigenous peoples. *Subst. Use Misuse* **32,** 1525–1538.
11. Blaszczynski, A., Huynh, S., Dumlao, V.J., and Farrell, E. (1998) Problem gambling within a Chinese speaking community. *J. Gambl. Stud.* **14(4),** 359–380.
12. Cultural Partners Australia Consortium (2000) The Impact of Gaming on Specific Cultural Groups. Victorian Casino and Gaming Authority, Victorian Commission for Gambling Regulation, Melbourne, Victoria.
13. Oei, T.P., Lin, J., and Raylu, N. (2006) The relationship between gambling cognitions, psychological states, and gambling: a cross-cultural study of Chinese and Caucasians in Australia. *J. Cross-Cultural Psychol.* **39(147),** 147–161.
14. Raylu, N. and Oei, T.P. (2004) Role of culture in gambling and problem gambling. *Clin. Psychol. Rev.* **23**, 1087–1114.

15. Wong, I.L.K. and So, E.M.T. (2003) Prevalence estimates of problem and pathological gambling in Hong Kong. *Am. J. Psychiatry* **160(7),** 1353–1354.
16. Taormina, R.J. (2009) Social and personality correlates of gambling attitudes and behaviour among Chinese residents of Macau. *J. Soc. Pers. Relationships* **26(8),** 1047–1071.
17. American Psychiatric Association (2000) *Diagnostic and Statistical Manual of Mental Disorders.* Vol. 4. Washington, D.C.
18. Loo, J.M., Raylu, N., and Oei, T.P. (2008) Gambling among the Chinese: a comprehensive review. *Clin. Psychol. Rev.* **28,** 1152–1166.
19. Wong, T.L.K. and So, E.M.T. (2003) Prevalence estimates of problem and pathological gambling in Hong Kong. *Am. J. Psychiatry* **160**, 1353–1354.
20. Petry, N.M. (2006) *Pathological Gambling: Etiology, Comorbidity, and Treatment*. American Psychological Association, Washington, D.C.
21. Mason, K. and Arnold, R. (2007) Problem gambling risk factors and associated behaviours and health status: results from the 2002/03 New Zealand Health Survey. *N. Z. Med. J.* **120(1257),** U2604. http://www.nzma.org.nz/journal/120-1257/2604/.
22. Ministry of Health (2007) Problem Gambling Intervention Services in New Zealand: 2006 Service-User Statistics. Public Health Intelligence, Monitoring Report No. 14. Wellington.
23. Jacques, C., Ladouceur, R., and Feriand, F. (2000) Impact of availability on gambling: a longitudinal study. *Can. J. Psychiatry* **45(9),** 810–815.
24. Price, J.A. (1972) Gambling in traditional Asia. *Anthropologica* **14(2),** 157–180.
25. Productivity Commission (2010) Gambling. Productivity Commission, Canberra, Australia.
26. Korn, D. and Shaffer, H. J. (1999) Gambling and the health of the public: adopting a public health perspective. *J. Gambl. Stud.* **15,** 289–365.
27. Lam, D. (2007) An observation study of Chinese Baccarat players. *UNLV Gaming Res. Rev. J.* **11**, 63–73.
28. Lanciani, R. (1892) Gambling and cheating in Ancient Rome. *North Am. Rev.* **155(428),** 97–105.
29. Kwok, S.L. and Shiu, Q.F. (1996) *History of Chinese Gambling*. Min-chin, Taipei, Taiwan.
30. Rayment, C. (1950) Anti-gambling measures. *Classical Wkly.* **43(8),** 121–122.
31. Lam, D. (2009) Unlocking the world of Chinese gambling. *Global Gaming Bus.* **8(9).** Retrieved February 27, 2010 from http://ggbmagazine.com/issue/vol-8-no-9-september-2009.
32. Barnett, C. (1999) The last outpost. *Hist. Today* **49(12),** 38–39.
33. Costello, J.G., Hallaran, K., Warren, K., and Akin, M. (2008) The luck on Third Street: archaeology of Chinatown, San Bernardino, California. *Hist.l Archaeol.* **42(3),** 136–151.
34. Shek, D.T.L. and Chan, E.M.L. (2009) Assessment of problem gambling in a Chinese context: the Chinese G-MAP. *TheScientificWorldJOURNAL: TSW Holistic Health & Medicine* **9**, 548–556.
35. Oei, T.P., Lin, J., and Raylu, N. (2008) The relationship between gambling cognitions, psychological states, and gambling: a cross-cultural study of Chinese and Caucasians in Australia. *J. Cross-Cultural Psychol.* **39(2),** 147–161.
36. Bagby, R.M., Vachon, D.D., Bulmash, E.L., Toneatto, T., Quilty, L.C., and Costa, P.T. (2007) Pathological gambling and the five-factor model of personality. *Pers. Individ. Differences* **43(4),** 873–880.
37. Myrseth, H., Pallesen, S., Molde, H., Johnsen, B.H., and Lorvik, I.M. (2009) Personality factors as predictors of pathological gambling. *Pers. Individ. Differences* **47(8),** 933–937.
38. Anderson, K. (1997) Gambling. Probe Ministry. Retrieved June 29, 2010 from http://www.leaderu.com/orgs/probe/docs/gambling.html.
39. Pontell, H. and Geis, G. (2008) Religion and the psychology of gambling in China and the US. In *Psychology of Gambling*. Esposito, M.J., Ed. Nova Biomedical Books, Hauppauge, N.Y. pp. 1–14.
40. Wang, Z. (2007) Two Decades of China's Lottery. Retrieved February 27, 2010 from http://www.china.org.cn/english/China/222227.htm.
41. China Internet Information Center. Sports Lottery. Retrieved March 1, 2010 from http://www.china.org.cn/english/features/China2004/107178.htm.
42. Li, X. Gambling still a fact of Chinese life. *China Daily*. Online on April 4, 2009. Retrieved March 23, 2010 from http://www.chinadaily.com.cn/bw/2009-04/27/content_7717886.htm.
43. Wikipedia, the free encyclopedia. Gambling in Macau. Retrieved February 27, 2010 from http://en.wikipedia.org/wiki/Gambling_in_Macau.
44. van Schalkwyk, G.J., Tran, E., and Chang, K. (2006) The impact of Macao's gaming industry on family life. *China Perspectives*, **64**, March–April. Online on April 1, 2009. http://chinaperspectives.revues.org/603.
45. The Hong Kong Jockey Club. About HKJC. Retrieved February 27, 2010 from http://www.hkjc.com/english/corporate/corp_about.asp.
46. World Gambling Review. Hong Kong Gambling. Retrieved July 31, 2010 from http://www.worldgamblingreview.com/gambling/hong-kong/.
47. The Hong Kong Jockey Club. Responsible Gambling Policy. Retrieved March 4, 2010 from http://www.hkjc.com/english/corporate/corp_rgp.asp.
48. Sui, C. Taiwan approves gambling enclaves. *BBC News* on 12 January 2009. Retrieved March 4, 2010 from http://news.bbc.co.uk/2/hi/7823825.stm.

49. Wong, S. (2009) Greater China: Beijing losing the gambling battle. *Asia Times.* Online on June 27, 2009. Retrieved July 31, 2010 from http://www.atimes.com/atimes/China/KF27Ad01.html.
50. Tse, S., Wong, J., and Kim, H. (2004) A public health approach for Asian people with problem gambling in foreign countries. *J. Gambl. Issues* **12** (16 pages). Retrieved January 20, 2005 from http://www.camh.net/egambling/issue12/jgi_12_tse.html.
51. Chen, S. and Davenport, D. (2005) Cognitive-behavioral therapy with Chinese American clients: cautions and modification. *Psychother. Theory Res. Pract. Train.* **42(1),** 101–110.
52. Nishio, K. and Bilmes, M. (1987) Psychotherapy with Southeast Asian American clients. *Prof. Psychol. Res. Pract.* **18(4),** 342–346.
53. Wong, E., Beutler, L., and Zane, N. (2007) Using mediators and moderators to test assumptions underlying culturally sensitive therapies: an exploratory example. *Cultur. Divers. Ethnic Minor. Psychol.* **13(2),** 169–177.
54. Shek, D.T.L. (2010) Positive youth development and behavioral intention to gamble among Chinese adolescents in Hong Kong. *Int. J. Adolesc. Med. Health* **22(1),** 163–172.
55. Shek, D.T.L. and Lee, J.J. (2010) Prevention of problem gambling in Chinese adolescents: relevance of problem gambling assessment and positive youth development frameworks. *Int. J. Adolesc. Med. Health* **22(1),** 139–151.
